# Intranasal Sendai Virus Vaccination of Seropositive Children 1 to 2 Years of Age in a Phase I Clinical Trial Boosts Immune Responses Toward Human Parainfluenza Virus Type 1

**DOI:** 10.3390/vaccines13040430

**Published:** 2025-04-19

**Authors:** Elisabeth Adderson, Kim J. Allison, Kristen Branum, Robert E. Sealy, Bart G. Jones, Sherri L. Surman, Rhiannon R. Penkert, Randall T. Hayden, Charles J. Russell, Allen Portner, Karen S. Slobod, Julia L. Hurwitz

**Affiliations:** 1 Department of Infectious Diseases, St. Jude Children’s Research Hospital, 262 Danny Thomas Place, Memphis, TN 38105, USA; elisabeth.adderson@stjude.org (E.A.); kim.allison@stjude.org (K.J.A.); kristen.branum@stjude.org (K.B.); bart.jones@stjude.org (B.G.J.); sherri.surman@stjude.org (S.L.S.); rpenkert@uoregon.edu (R.R.P.); charles.russell@stjude.org (C.J.R.); cambridgeconsultingid@gmail.com (K.S.S.); 2 Department of Chemistry and Biochemistry, Institute of Molecular Biology, University of Oregon, Eugene, OR 97403, USA; 3 Department of Pathology, St. Jude Children’s Research Hospital, Memphis, TN 38105, USA; randall.hayden@stjude.org; 4 Department of Host-Microbe Interactions, St. Jude Children’s Research Hospital, Memphis, TN 38105, USA; 5 Cambridge ID & Immunology Consulting LLC., Sommerville, MA 02144, USA

**Keywords:** Sendai virus, intranasal, vaccine, pediatric

## Abstract

Background/Objectives: Human parainfluenza virus type 1 (hPIV-1) is a major cause of serious respiratory diseases in young children. Annually, hPIV-1 results in approximately 10,000 hospitalizations in the United States due to croup, bronchiolitis, and/or pneumonia, and 10,000 deaths worldwide due to acute lower respiratory tract infections among children less than 5 years of age. Despite the burden of disease, no vaccine for hPIV-1 is currently approved. Sendai virus (SeV) is a murine PIV-1. It has structural similarities with hPIV-1 and is currently under clinical development as an hPIV-1 Jennerian vaccine. Attributes of SeV include the following: (a) needleless delivery, (b) rapid and durable serum antibody responses after a single intranasal administration, (c) durable IgG and IgA responses in the nasal mucosa, and (d) use as a platform for recombinant vaccines against multiple pediatric pathogens. Evaluation of the tolerability, safety, and immunogenicity of intranasal SeV in healthy adults and seropositive children 3 to 6 years of age was previously conducted and supported vaccine advancement to evaluation in younger children. Methods: Three seropositive children 1 to 2 years of age received a single intranasal dose of 5 × 10^5^ EID_50_ SeV (SENDAI, Clinicaltrials.gov NCT00186927). Adverse events were collected for 28 days post-vaccine administration using diary cards and participants were followed for six months in total. Sera were collected longitudinally for clinical laboratory and virus-specific antibody tests. Nasal swabs were collected longitudinally for virus and mucosal antibody tests. Results: Intranasal SeV was well tolerated, with only mild grade 1–2 events that resolved spontaneously. No serious adverse events, medically attended adverse events, or adverse events causing protocol termination were reported. One participant had positive nasal swabs for inoculated SeV during the first week after vaccination. Although children had measurable PIV-1-specific serum antibodies at baseline, intranasal SeV vaccination resulted in significant serum antibody increases in all participants. Similarly, there were significant increases in PIV-1-specific nasal IgG and IgA levels in all participants. Elevated antibody levels persisted through the six months of follow-up. Conclusions: Intranasal SeV was well tolerated and uniformly immunogenic in seropositive children 1 to 2 years of age. Results encourage the further evaluation of SeV and SeV-based recombinants as potential intranasal vaccines for the prevention of infection by hPIV-1 and other serious respiratory pathogens.

## 1. Introduction

Respiratory viruses, including human parainfluenza viruses (PIVs), respiratory syncytial viruses (RSVs), and metapneumoviruses (MPVs), are among the leading causes of pediatric deaths worldwide [1,2,3,4,5,6,7,8,9,10]. Human parainfluenza virus type 1 (hPIV-1) is best known for causing serious laryngotracheobronchitis (croup) in young children, and also causes bronchiolitis and pneumonia [3,4,5,6]. Most children are first exposed to hPIV-1 before 5 years of age, and these first exposures are associated with the greatest risk of disease. Among children < 5 years of age, hPIV-1 is reported to result in approximately 10,000 U.S. hospitalizations due to croup, bronchiolitis, and/or pneumonia, and 10,000 acute lower respiratory tract infection (ALRI)-associated deaths worldwide annually [5,10].

Important advances in maternal vaccination and monoclonal antibody administration provide newborns with transient passive immunity against some respiratory viruses [11,12,13,14]. By coupling passive immunity strategies with pediatric vaccination programs, both transient and long-term protection may be achieved [15].

Currently, there is no licensed vaccine for hPIV-1 [4,16]. Clinical evaluation of a formalin-treated hPIV-1 vaccine was conducted over 50 years ago, and although there were no safety concerns (unlike those observed following the testing of a formalin-treated RSV vaccine), the inactivated hPIV-1 vaccine was not protective [17,18,19,20]. Another research strategy involved attenuation of hPIV-1 isolates. This strategy advanced to clinical trials, but researchers found that immunogenicity was compromised by virus attenuation [21].

This report summarizes a distinct approach to hPIV-1 vaccine design and clinical development, employing a method advanced by Dr. Edward Jenner in the late 1700s. Jenner was the first to demonstrate formally that material taken from a bovine lesion (cowpox) could be used as a human vaccine against smallpox [22]. While vaccine mechanisms were not understood at the time, it was ultimately realized that structural similarities between cowpox and smallpox viruses supported the induction of cross-reactive, protective immune responses. In the 1900s, the World Health Organization sponsored a vaccinia virus-based vaccination campaign that eradicated smallpox from the human population [23]. To date, no other vaccine program has achieved the success of the Jennerian approach [24,25,26].

The Jennerian strategy described in this report uses SeV, a PIV of mice, as a vaccine for hPIV-1. SeV has significant similarities in sequence and structure with hPIV-1 [4]. Both viruses are members of the family *Paramyxoviridae* [27]. Both viruses consistently induce long-lasting cross-reactive binding and neutralizing antibodies and T cells, allowing hPIV-1 to serve as a vaccine for SeV and allowing SeV to serve as a vaccine for hPIV-1 [4,28,29,30,31].

When SeV was first discovered, researchers thought the virus was derived from a human with respiratory disease. However, it was subsequently determined that clinical samples had been passaged through mice before virus isolation and that the murine virus SeV had been isolated inadvertently [32,33]. Additional research showed that SeV caused respiratory disease in rodents but not in primates, including macaques, African green monkeys, and chimpanzees [4,16,34].

One explanation for the safety of SeV in non-rodent species may relate to SeV’s C protein, which differs from that of hPIV-1 [35,36,37]. The C protein is non-essential but promotes anti-interferon (IFN) and anti-apoptosis activities required for hPIV-1 virulence in primates. In contrast to hPIV-1, SeV is sensitive to human IFN in human cells in late stages of infection [35].

Apart from its proven safety profile in primates, SeV is an attractive vaccine candidate because of its induction of rapid, potent, systemic, mucosal, and long-lasting B cell, CD4^+^ T cell, and CD8^+^ T cell immune responses after a single needleless delivery [4,28,29]. In small-animal studies, SeV induced durable virus-specific antibody-forming cells (AFCs), including IgA-producing cells, in nasal-associated lymphoid tissues and protected animals from hPIV-1 challenges just one week after vaccination [29].

By generating immune responses in both the upper and lower respiratory tract, the intranasal vaccine can stop the target pathogen at its point of entry. It is essential to limit viral spread before pathogens enter the lung, where the most serious diseases, including pathogenic hyperinflammatory responses associated with enhanced immunity, can occur [17,18,19,20].

In parallel with the development of SeV as a vaccine against hPIV-1, SeV recombinants have been produced using reverse genetics technology to serve as therapeutics [38,39] or to serve as vaccines against a variety of human pathogens [40,41,42,43,44,45,46]. As examples, recombinants were developed to express proteins from RSV (e.g., SeVRSV) [46,47], hPIV-2, hPIV-3, or human metapneumovirus. Vaccines were used individually or as cocktails to protect against several pathogens simultaneously [4,46]. In small research animals, SeV-based vaccines were successful in the context of passive immunity [48] and at doses as low as 10^2^ egg-infectious doses (EID_50_) [4].

A phase I clinical trial of SeV has already tested the vaccine in adults and children 3 to 6 years of age (protocol SENDAI). The pediatric study demonstrated that a single dose of intranasal SeV was sufficient to increase hPIV-1-specific binding and neutralizing serum antibodies, even when study participants were seropositive at baseline. SeVRSV has also been tested clinically in adults [4,49].

We now report the results of SeV vaccination in younger children. This study was designed (i) to safety test a dose of 5 × 10^5^ EID_50_ SeV administered intranasally in participants 1 to 2 years of age, and (ii) to determine if the 5 × 10^5^ EID_50_ dose of SeV could boost antibody responses toward hPIV-1, both systemically and mucosally, in the presence of pre-existing immunity in young children. Results demonstrated that intranasal SeV was well tolerated and uniformly immunogenic in seropositive children 1 to 2 years of age.

## 2. Materials and Methods

### 2.1. Study Protocol

The SENDAI study was conducted at St. Jude Children’s Research Hospital (St. Jude), Memphis, TN, under a U.S. IND following review by the U.S. FDA. Approval was received from the St. Jude IRB (3/30/04, protocol designation: SENDAI). This phase I protocol was registered in Clinicaltrials.gov (NCT00186927, registration submission 9/12/05). The study of vaccinated children between 1 and 2 years of age occurred within years 2015–2018. Informed consent was obtained from the parents/guardians of all children involved in the study. Investigations were conducted in accordance with the principles outlined in the Declaration of Helsinki.

Three children between 1 and 2 years of age were vaccinated with SeV. Among the eligibility requirements were (i) clinical laboratory tests within normal values, (ii) a positive test for pre-existing PIV-1-specific serum antibodies, and (iii) no other immunizations within 30 days of receiving the study vaccine. Among the exclusion criteria were (i) history of egg allergy, (ii) history of lung disease, asthma, hospitalization for respiratory illness, immunodeficiency, or any serious underlying illness or condition, (iii) expected same-room contact with an immunodeficient individual or a child less than 24 months of age within 14 days of vaccination or before evidence of vaccine clearance, and (iv) current use of investigational or immunosuppressive drugs, antibiotics, or antivirals.

Pre-existing PIV-1-specific serum antibodies were measured at the pre-screen visit with an SeV-based enzyme linked-immunosorbent assay (ELISA) to ensure the presence of vaccine-specific immunity at baseline. Vaccine was given as a single dose of 5 × 10^5^ EID_50_ diluted in saline, 0.25 mL per nostril. Study site visits were conducted in the clinic on days 2, 4, 7, 14, 28, and 182. Phone calls were made to parents/guardians on days 1, 3, 5, 6, once between days 7 and 14, and twice between days 14 and 28. Parents/guardians were provided diary cards at the time of vaccination to record all adverse events using free text for each day from inoculation to day 28 post-inoculation. The duration and intensity (grades 1–4) of events were collected. Parents/guardians were asked to report any adverse events after day 28 at the 6 month visit.

Blood was obtained at screening and on days 14, 28, and 182 for clinical safety laboratory testing and measurement of virus-specific antibody levels. Nasal swabs were obtained at screening and on days 2, 4, 7, 14, and 28 for the testing of virus and virus-specific antibodies.

### 2.2. Vaccine

Unmodified, live SeV (Enders strain) was propagated in chick egg allantoic fluid. Hens’ eggs were purchased from Spafas, Inc., Preston, CT. Virus was purified from allantoic fluid on a sucrose cushion followed by a sucrose gradient. Purified virus was stored frozen in buffer and stabilizer at −80 °C. Prior to use, the vaccine was thawed and diluted > 1:50 in sterile saline for intranasal inoculation. Product tests included assays for sterility, adventitious agents, DNA, protein, and endotoxin. Because the vaccine was derived from eggs and possibly retained residual egg allergens, an exclusion criterion for participant study entry was a history of egg allergy.

### 2.3. Tests for SeV and Human Pathogens in Nasal Swabs

Nasal samples were tested for PIV-1 in the CLIA-certified Clinical Microbiology Laboratory at St. Jude by a 21-day viral culture or by molecular testing. If PIV-1 was detected, further research tests were used to confirm SeV identity. Briefly, nasal samples were inoculated into allantoic cavities of 10-day-old embryonated hens’ eggs at 35 °C for 72 h and tested for hemagglutination with chicken red blood cells. Positive allantoic fluids were amplified in four-day LLC-MK_2_ cell [50] cultures. Cell monolayers were then fixed with acetone/PBS (80%/20%), air-dried, rehydrated with PBS, and blocked. An ELISA was performed to discriminate between SeV and hPIV-1 using a mouse monoclonal antibody, S2, that bound SeV and not hPIV-1. The assay was developed with goat anti-mouse IgG-HRP (SBA), TMB (KPL, Gaithersburg, MD, USA), and stop solution, (1M H_3_PO_4_), after which plates were read (OD_450_ nm).

If an adverse event was identified and considered possibly related to the vaccine, PCR amplification (Biofire Respiratory 2.1 Panel, Biomerieux, Salt Lake City, UT, USA) was performed to test for human respiratory pathogens in nasal samples.

### 2.4. Measurement of Serum and Nasal Antibody Levels

ELISA methodologies have been described previously [49]. Briefly, preparations of both SeV and hPIV-1 were produced to support respective tests of SeV- and hPIV-1-specific antibodies. Each virus was purified and placed in disruption buffer (0.005 M Tris pH 7.8, 0.06 M KCl, 0.05% Triton X-100) for 5 min at room temperature. Virus was then diluted with PBS at least 1:10 for plating at 1 µg/mL in ELISA plates for overnight incubation at 4 °C. Plates were then washed 3X with PBS and blocked with 100 µL/well 3% bovine serum albumin (BSA) in PBS for a 1–2 hr incubation at RT or an overnight incubation at 4 °C. Serum or nasal samples were added to blocked plates at 50 µL/well in triplicate using serial dilutions. Specifically, serum samples were diluted 1:1000 and then serially diluted 1:10 in 3% BSA and 0.1% Tween-20 in PBS, while nasal samples were diluted 1:5, 1:25, and 1:125 in the same buffer. After at least 1 h, plates were washed 7X with 0.1% tween-20 in PBS and developed with either anti-human IgG or anti-human IgA conjugated to alkaline phosphatase, followed by p-nitrophenyl phosphate. Plates were read (OD 405 nm). *T*-tests were performed with Excel software.

## 3. Results

### 3.1. SeV Vaccinations

Three participants, including two males and one female between 1 and 2 years of age, received intranasal SeV. Participants were black or black/white and between the ages of 16 and 23 months. Nasal swabs were collected at screening, and on days 2, 4, 7, 14, and 28 post-vaccination to test for the presence of SeV. SeV was only detected in one participant (designated #2). Positive tests were on days 2, 4, and 7, and the virus was undetectable on later days.

### 3.2. Safety Data

Safety laboratory parameters were all within normal limits for all three participants. No participant reported any grade 3 or 4 adverse event. Two of the three participants reported adverse events in the 28 days post-inoculation; all were grade 1 or 2 in severity and all resolved spontaneously. One participant (designated #1) reported grade 1 fever and cough on day 8 and grade 1 irritability and grade 2 diarrhea on day 9. Fever resolved within one day (day 9); irritability and diarrhea resolved by day 14, and cough resolved by day 16. Participant #2 reported only grade 1 rhinorrhea (day 5 that resolved on day 7, day 11 that resolved on day 12, and day 20 that resolved on day 28). Throughout the study, there were no reported serious adverse events, medically attended adverse events, or adverse events causing protocol termination.

### 3.3. Immunogenicity

**Figure 1:**
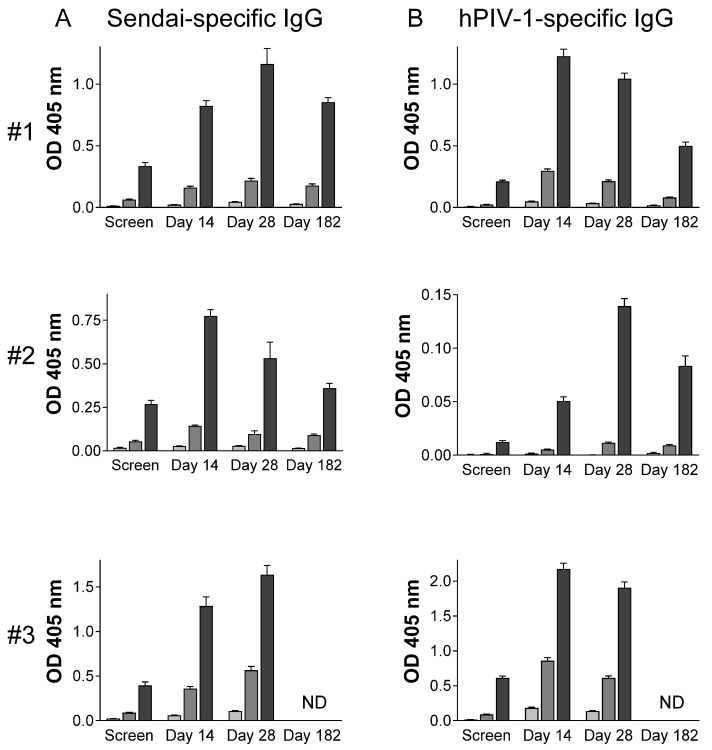
SeV- and hPIV-1-specific serum antibodies. Serum antibody ELISAs were performed with plates coated with SeV (**A**) or hPIV-1 (**B**) and samples diluted 1:1,000 (black bars), 1:10,000 (dark grey bars), and 1:100,000 (light grey bars). Results are shown for the three participants (#1 to #3). Means and standard errors are shown. Participant #3 did not return for the day 182 visit. ND = not done.

**Figure 2:**
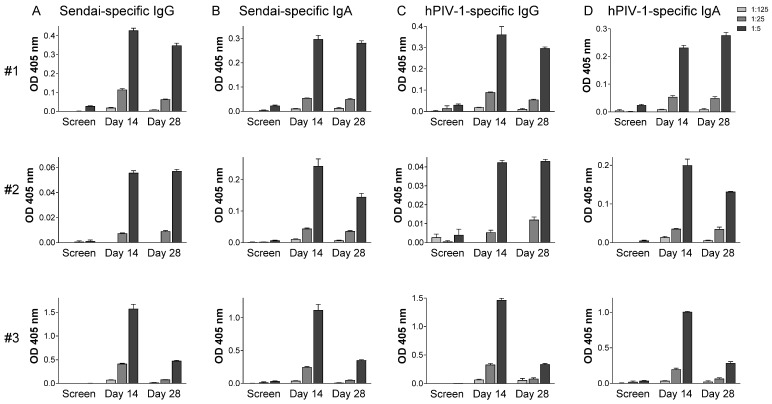
SeV- and hPIV-1-specific antibodies in the upper respiratory tract. ELISAs were with nasal samples diluted serially 1:5 (black bars), 1:25 (dark grey bars), and 1:125 (light grey bars) and with SeV-coated (**A**,**B**) or hPIV-1-coated (**C**,**D**) plates. Means and standard errors are shown for three participants (#1 to #3).

Tests were performed to measure improvements in PIV-1-specific antibody responses in sera and in the upper respiratory tract of these seropositive children. As shown in Figure 1, all participants responded with increased serum antibody levels. SeV- and hPIV-1-specific antibody increases from baseline (based on absorbance readings for the highest sample concentrations, 1:1000) were between 2.9- and 11.3-fold among participants. Peaks were observed on day 14 or 28. As shown in Figure 2, SeV- and hPIV-1-specific IgG and IgA antibodies in nasal samples were also increased after SeV vaccination in every participant. Antibody increases from baseline (based on absorbance readings for the highest sample concentrations, 1:5) were between 9.5- and 525.7-fold. All increases were statistically significant when pre-and post-vaccine samples were compared using the highest sample concentrations (*T* test, *p* < 0.05).

Altogether, results showed that a dose as low as 5 × 10^5^ EID_50_ SeV was well tolerated and reliably increased immune responses in each of the tested seropositive participants.

## 4. Discussion

This is the first study to test SeV as an intranasal vaccine against hPIV-1 in seropositive children between the ages of 1 and 2 years. The SeV dose of 5 × 10^5^ EID_50_ was well tolerated and significantly increased systemic and mucosal antibody responses in each of the three study participants. Both IgG and IgA responses were increased in the upper respiratory tract. Our previous study of SeV vaccination in older seropositive children demonstrated that the upregulated PIV-1-specific antibodies bound and neutralized hPIV-1 [49].

### 4.1. Vaccine-Induced Immune Responses in Systemic and Mucosal Tissues

Previous small-animal studies have demonstrated rapid and durable protection induced by SeV vaccines [28,29]. Virus-specific AFCs and T cells were increased, not just in peripheral blood and bone marrow, but in the upper and lower respiratory tract [28], providing a formidable immune barrier against the pathogen at its point of entry. Intranasal vaccines are unlike intramuscular vaccines, the latter of which can recruit AFCs well to the bone marrow and lung, but not to nasal passages [51]. The AFCs induced by SeV express both virus-specific IgG and IgA in nasal-associated lymphoid tissues [29]. Because IgA can transcytose respiratory epithelial cells, it is best positioned for respiratory tract immune surveillance [52]. Like B cells, SeV-induced T cells are resident both in the blood and mucosa for extended periods. Together, B cell and T cell effectors recognize and neutralize hPIV-1 [4]. By blocking the pathogen at its point of entry, a robust SeV-induced immune response in the upper respiratory tract may prevent the pathogen from trafficking into lower respiratory tract tissues where tissue damage and inflammation can cause considerable morbidity and mortality [17,18,19,20]. Small-animal data predicted the success described in this report of the SeV vaccine in children.

While peaks of virus-specific serum antibodies were at 14 or 28 days, antibodies remained detectable at the 6-month time point. It is noteworthy that establishing correlates of protection for vaccines based on virus-specific serum antibodies alone can be difficult. Even when serum antibody levels wane after effective vaccination, AFCs continue to secrete antibodies and memory cells are poised for rapid recall upon antigen re-exposure, thereby assisting durable protection [53,54,55,56,57,58].

### 4.2. SeV Immunogenicity in the Context of Pre-Existing Immunity

Maternal vaccines and monoclonal antibodies are being developed to delay infections during an infant’s first months of life, particularly in the RSV and influenza virus fields. However, when antibodies are unavailable or when passively transferred antibodies decay [12,14], virus susceptibility increases. Pediatric vaccinations are then desired to provide long-term protection.

In a pre-clinical study, a model was developed to test vaccines in the context of passively transferred antibodies. Briefly, virus-specific monoclonal or polyclonal antibodies were administered to adult cotton rats prior to vaccination. Results showed that SeV-based vaccines remained immunogenic in this context [48], again predicting the success of SeV in seropositive children 1 to 2 years of age. The passively transferred antibodies and SeV-based vaccines functioned in parallel to prevent infections of the respiratory tract both transiently and durably. This finding paves the way for joint usage of passively transferred antibodies and vaccination in young children.

### 4.3. Limitations

This study was limited by its small participant number, its performance in a single geographical site, the testing of only one vaccine dose, and a focus on antibody upregulation systemically and mucosally. The virus was grown in hens’ eggs, causing exclusion of individuals with histories of egg allergies. The exclusion of participants with egg allergies may be remedied in the future by the production of virus vaccines in mammalian tissue culture cells [59,60].

### 4.4. Future Prospects

The overall tolerability and immunogenicity of 5 × 10^5^ EID_50_ SeV in children aged 1 to 2 years supports advancing the vaccine to studies in seronegative children. Given that a vaccine dose of 10^2^ has been demonstrated effective in pre-clinical models [4], it is anticipated that clinical studies in seronegative children may initiate with doses considerably less than 5 × 10^5^ EID_50_. SeV vaccines may be tested either alone or in parallel with passive immune strategies [48] to ensure that young children are continuously protected from PIV infections.

SeV recombinants remain promising vehicles for protection against pathogens other than hPIV-1 and for the general delivery of preventive and therapeutic proteins to children. Using reverse genetics, SeV recombinants have already been produced to target a variety of viral pathogens, including hPIV-3, MPV, and RSV. Both individual and cocktail vaccines have proven successful in pre-clinical models, protecting against as many as four different viruses at once [4]. The demonstration of SeV’s tolerability and capacity to upregulate local and systemic immune responses in this pediatric study completes an important step in leveraging SeV as a vaccine platform.

## 5. Conclusions

SeV was tested as an intranasal vaccine in seropositive children 1 to 2 years of age. Results illustrated the tolerability and immunogenicity of live, unmodified SeV, and demonstrated increases in systemic and mucosal antibody responses despite the pre-existing seropositivity of study participants. Data encourage the progression of clinical trials to test SeV and SeV-based recombinants in seronegative children, the children who are most in need of PIV-1 vaccines.

## Data Availability

Data are provided in the manuscript, and additional details will be provided upon request from authors.
